# Supplementary material for: Predicting structures of large protein assemblies using combinatorial assembly algorithm and AlphaFold2
Source: bioRxiv. 2023 May 16:2023.05.16.541003. Preprint. [Version 1] doi: 10.1101/2023.05.16.541003 (PMC10245790; doi:10.1101/2023.05.16.541003)
Supplement: Supplement 1 [file NIHPP2023.05.16.541003v1-supplement-1.pdf]

bioRxiv preprint doi: https://doi.org/10.1101/2023.05.16.541003; this version posted May 16, 2023. The copyright holder for this preprint (which was not certified by peer review) is the author/funder, who has granted bioRxiv a license to display the preprint in perpetuity. It is made available under aCC-BY 4.0 International license.

Supplementary Materials

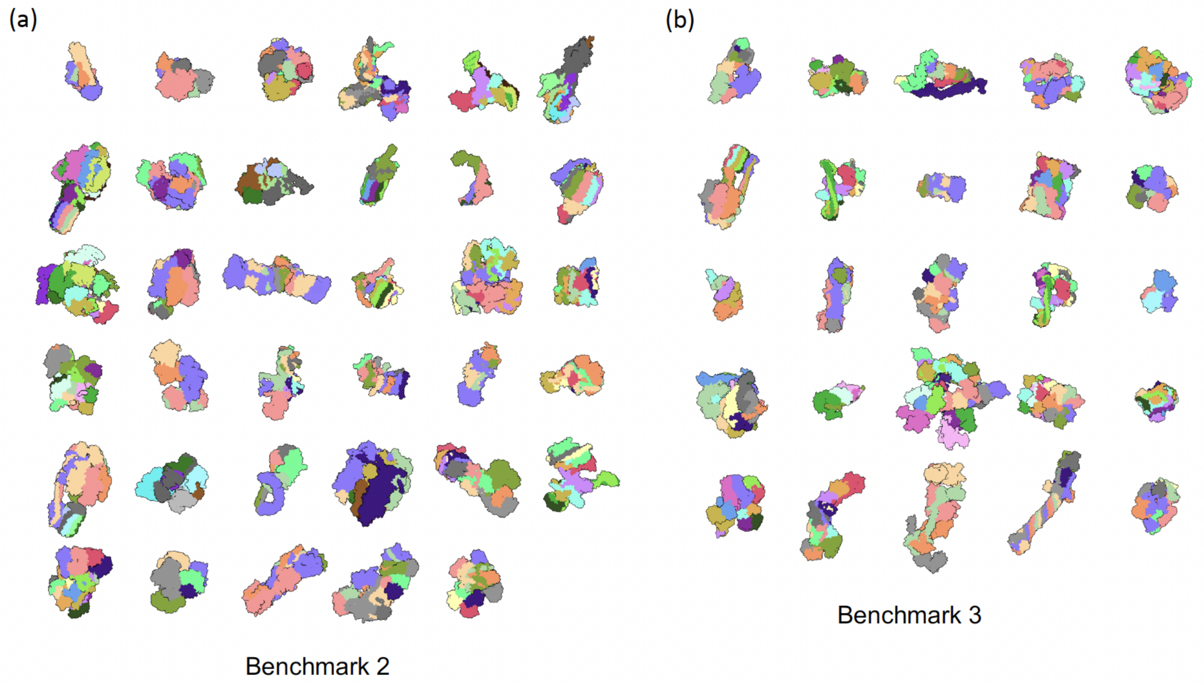

Figure S1. Heteromeric complexes (colored by chain) from **(a)** Benchmark 1 and **(b)** Benchmark 2.

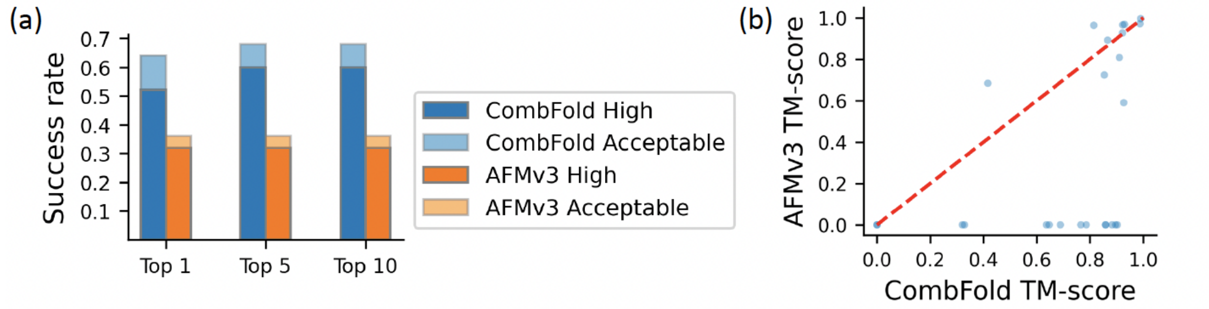

Figure S2. Accuracy of CombFold on Benchmark 2. (a) The Top-N (N=1, 5, 10) success rate of CombFold (blue) and AFMv3 (orange). **(b)** TM-score of AFMv3 models vs. CombFold models for Top-5 results.

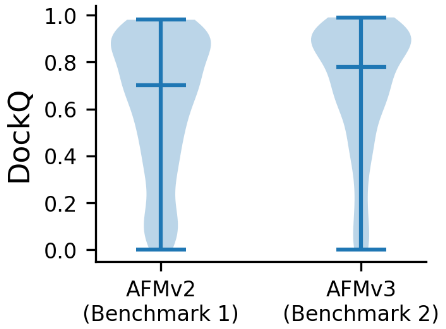

bioRxiv preprint doi: https://doi.org/10.1101/2023.05.16.541003; this version posted May 16, 2023. The copyright holder for this preprint (which was not certified by peer review) is the author/funder, who has granted bioRxiv a license to display the preprint in perpetuity. It is made available under aCC-BY 4.0 International license.

Figure S3. Accuracy of pairwise predictions for AFMv2 and AFMv3. DockQ scores of pairwise interactions predicted by AFM on Benchmark 1 (AFMv2) and Benchmark 2 (AFMv3), for which the PAE-based score is over 50. The median score is 0.70 and 0.78 for AFMv2 and AFMv3, respectively.

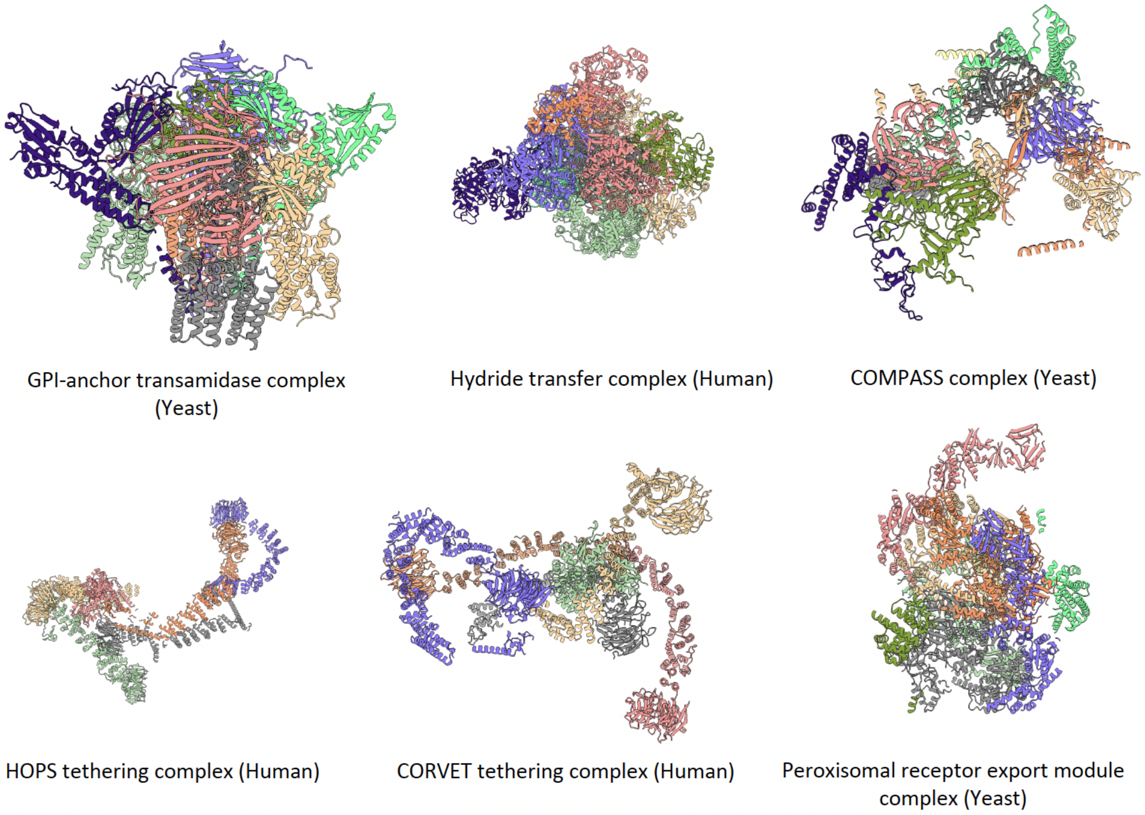

Figure S4. Predicted complexes from Complex Portal with High or Medium confidence.

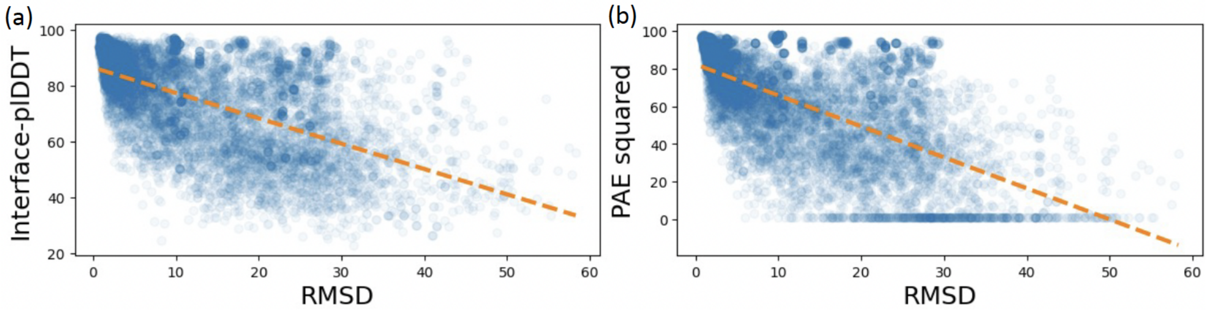

Figure S5. Comparison of average interface plDDT and PAE. (a) Interface-plDDT vs. pairwise RMSD (Pearson r = 0.59). (b) PAE vs. pairwise RMSD (Pearson r = 0.64).

bioRxiv preprint doi: https://doi.org/10.1101/2023.05.16.541003; this version posted May 16, 2023. The copyright holder for this preprint (which was not certified by peer review) is the author/funder, who has granted bioRxiv a license to display the preprint in perpetuity. It is made available under aCC-BY 4.0 International license.

Chain	Group 1	Group 2	Group 3	Group 4
A		1-175	155-320	300-409
B		1-154	134-302	282-363
C		1-155	135-300	280-384
D		1-167	147-315	295-487
E		1-174	154-318	298-410
F		1-157	137-300	280-423
H+I	All	All		
G+J	All			

Table S1: Subdivision of H1137 into groups of subunits with overlapping domains. Each cell lists the amino acids indexes of the chain present in each model. The division was made so that the helical domain in chains A-F will be calculated as a group (Model 3).

Gene	Change	Structure position
(P) Elp1	Arg696Pro	near interface with Elp6
(L) Elp1	Pro914Leu	interface with Elp2
(P) Elp2	His206Arg	interface with Elp1/Elp3
(P) Elp2	Arg462(Gln/Leu)	core
(P) Elp2	Thr555Pro	core
(L) Elp2	Thr405Ile	core
(P) Elp4	Arg289Trp	interface with Elp6
(L) Elp4	Tyr91Cys	interface with Elp6
(L) Elp4	Leu296Ile	core
(L) Elp6	Leu118Trp	near interface with Elp5

Table S2: ClinVar missense mutations for Elongator holoenzyme complex labeled as pathogenic (P) or likely pathogenic (L).
